# Comment on Rosenzweig et al. Very Low Prostate PET/CT PSMA Uptake May Be Misleading in Staging Radical Prostatectomy Candidates. *J. Pers. Med.* 2022, *12*, 410

**DOI:** 10.3390/jpm12050800

**Published:** 2022-05-16

**Authors:** Hans Veerman, André N. Vis, Maarten Donswijk, Henk G. van der Poel

**Affiliations:** 1 Department of Urology, Netherlands Cancer Institute-Antoni van Leeuwenhoek Hospital, 1066 CX Amsterdam, The Netherlands; h.vd.poel@nki.nl; 2 Department of Urology, Amsterdam University Medical Centres, Location VU Medical Centre, 1081 HV Amsterdam, The Netherlands; a.vis@amsterdamumc.nl; 3 Prostate Cancer Network Netherlands, 1066 CX Amsterdam, The Netherlands; 4 Department of Nuclear Medicine, Netherlands Cancer Institute-Antoni van Leeuwenhoek Hospital, 1066 CX Amsterdam, The Netherlands; m.donswijk@nki.nl

With interest, we read the article by Rosenzweig et al. on the comparison of prostate cancer (PCa) patients with a low and high prostate-specific membrane antigen (PSMA) expression on positron emission tomography/computed tomography (PET/CT) [1]. Two names were used for the study group: ‘negative prostate PSMA PET/CT’ and ‘(very) low prostate PSMA uptake’. We believe these names represent two distinct entities and should not be interchanged. Negative PSMA PET/CT suggests the complete absence of PSMA expression in the PCa lesion, whereas low versus high PSMA uptake/expression is based on the quantitative assessment (SUVmax) of the visible PCa lesion. 

Rosenzweig et al. found that 54% of their cohort had PSMA-negative PCa. This is approximately 10 times higher than the proportion of PSMA-negative PCa found in other studies, which is inherent to their definition of a negative PSMA PET/CT, namely SUVmax ≤ 6.6 [2]. The choice for this cutoff is questionable. It was based on the patient with the highest benign PSMA expression in a different study. Uprimny et al. found that benign prostate tissue had a maximum SUVmax of 6.6 (median 3.9, minimum 2.5) [3]. The literature offers better alternatives. For example, Jiao et al. examined whether the SUVmax could distinguish between benign and malignant prostate tissue [4]. They found a cutoff value of 5.3 in a training set of 135 patients. In the prospective validation cohort (N = 58), the cutoff value achieved a sensitivity of 83%, a specificity of 81%, a PPV of 92%, a NPV of 65%, and an accuracy of 83% to detect prostate cancer with biopsy. Nevertheless, SUVmax values can only be compared when the patients underwent scans on the same PET system with an identical protocol (i.e., in compliance with EARL standards) using the same tracer [5]. The multicentre aspect of this study warrants that the results should be interpreted with caution. 

Another reason why PSMA-negative is not the correct term is that PSMA-negative patients had a visible lesion on PSMA PET/CT. It is somewhat contradictory that PCa lesions with absent PSMA expression have a median size of 12 mm on PSMA PET/CT. Therefore, the PSMA-negative cohort appears to consist, at least in part, of patients with low PSMA expression. We suggest reserving the term PSMA-negative or non-PSMA-expressing PCa for lesions with equal or lower PSMA expression than the surrounding prostate background of the same patient, as suggested by the E-PSMA guidelines [2,6]. Of course, this critique is of a purely semantic nature.

We commend Rosenzweig et al. on their efforts to study patients from five different centres. In line with the literature, they found that patients with a higher PSMA expression have prognostically less favorable outcomes. Bodar et al. reported that a higher SUVmax is associated with higher Gleason scores, higher pT-stage and lymph node metastases [7]. In a cohort of 848 patients, Roberts et al. showed that the SUVmax is even a predictor for biochemical recurrence after RARP, independent of other prognostic factors (i.e., Gleason score, pT-stage and positive surgical margins) [8]. Therefore, the SUVmax appears to be a novel biomarker for PCa outcomes.

When we compare the low PSMA expression cohort of Rosenzweig to a truly PSMA-negative cohort, we see that a similar proportion of patients have pT3 disease (46% vs. 60%), but the proportion of ISUP 5 disease was higher in the PSMA-negative cohort (3.8% vs. 27%), suggesting that PSMA-negative PCa is a different entity. We encourage the authors to publish the oncological outcomes of the cohort in relation to the SUVmax.
